# The p21-Dependent Radiosensitization of Human Breast Cancer Cells by MLN4924, an Investigational Inhibitor of NEDD8 Activating Enzyme

**DOI:** 10.1371/journal.pone.0034079

**Published:** 2012-03-22

**Authors:** Dong Yang, Mingjia Tan, Gongxian Wang, Yi Sun

**Affiliations:** 1 Division of Radiation and Cancer Biology, Department of Radiation Oncology, University of Michigan, Ann Arbor, Michigan, United States of America; 2 Department of Urology, The First Affiliated Hospital of Nanchang University, Nanchang, China; Penn State Hershey Cancer Institute, United States of America

## Abstract

Radiotherapy is a treatment choice for local control of breast cancer. However, intrinsic radioresistance of cancer cells limits therapeutic efficacy. We have recently validated that SCF (SKP1, Cullins, and F-box protein) E3 ubiquitin ligase is an attractive radiosensitizing target. Here we tested our hypothesis that MLN4924, a newly discovered investigational small molecule inhibitor of NAE (NEDD8 Activating Enzyme) that inactivates SCF E3 ligase, could act as a novel radiosensitizing agent in breast cancer cells. Indeed, we found that MLN4924 effectively inhibited cullin neddylation, and sensitized breast cancer cells to radiation with a sensitivity enhancement ratio (SER) of 1.75 for SK-BR-3 cells and 1.32 for MCF7 cells, respectively. Mechanistically, MLN4924 significantly enhanced radiation-induced G2/M arrest in SK-BR-3 cells, but not in MCF7 cells at early time point, and enhanced radiation-induced apoptosis in both lines at later time point. However, blockage of apoptosis by Z-VAD failed to abrogate MLN4924 radiosensitization, suggesting that apoptosis was not causally related. We further showed that MLN4924 failed to enhance radiation-induced DNA damage response, but did cause minor delay in DNA damage repair. Among a number of tested SCF E3 substrates known to regulate growth arrest, apoptosis and DNA damage response, p21 was the only one showing an enhanced accumulation in MLN4924-radiation combination group, as compared to the single treatment groups. Importantly, p21 knockdown via siRNA partialy inhibited MLN4924-induced G2/M arrest and radiosensitization, indicating a causal role played by p21. Our study suggested that MLN4924 could be further developed as a novel class of radiosensitizer for the treatment of breast cancer.

## Introduction

The SCF E3 ubiquitin ligases consist of Skp1, cullins, F-box proteins, and a RING protein, RBX or ROC. By promoting the ubiquitination and degradation of various key regulatory proteins, SCF E3 ligases control several important biological processes including cell cycle progression, signal transduction and DNA replication [1], [2]. While the substrate specificity of SCF E3s is determined by the F box proteins that bind to Skp1 and Cullins through its F-box domain and to substrates through its WD40 or leucine rich region domains [3], [4], the activity of SCF E3 ubiquitin ligases requires 1) the RING protein RBX or ROC that binds to E2 and facilitates ubiquitin transfer from E2 to substrates [5] and 2) cullin neddylation, which disrupts inhibitory binding by CAND1 [6], [7], [8], [9]. Accumulated evidence strongly suggests that abnormal regulation of SCF E3 ubiquitin ligases contributes to uncontrolled proliferation, genomic instability, and cancer [2], and SCF E3 ligases appear to be an attractive anticancer targets [10], [11], [12].

Although no specific small molecule inhibitor of E3 ubiquitin ligases has been successfully discovered and developed [13], [14], MLN4924, a potent small molecule inhibitor of Nedd8 activating enzyme (NAE) has demonstrated an inhibitory activity against SCF E3 ligases via inhibiting cullin neddylation [15]. Mechanistically, MLN4924 binds to NAE to create a covalent Nedd8-MLN4924 adduct that blocks NAE enzymatic activity [16]. By inactivating SCF E3 ubiquitin ligases, MLN4924 caused the accumulation of a number of SCF E3 substrates to induce apoptosis [15], [17], [18] and senescence [19], [20], [21], thus inhibiting tumor growth both *in vitro* and *in vivo*. MLN4924 has been advanced to several Phase I clinical trials [22].

We have recently shown that inactivation of SCF E3 ubiquitin ligases by siRNA knockdown of either RBX1 or RBX2 (also known as SAG, Sensitive to Apoptosis Gene) sensitized human cancer cells to radiation [23], [24], [25], and that Sag knockout also sensitized mouse embryonic stem cells to radiation via inducing apoptosis [26]. Most recently, we found that MLN4924 could act as a novel radiosensitizing agent by causing accumulation of CDT1 and WEE1 to enhance radiation-induced DNA damage, aneuploidy, G2/M arrest and apoptosis in pancreatic cancer cells [27]. Here we extended our MLN4924-radiosensitization study from human pancreatic cancer cells to breast cancer cells and reported that MLN4924 is a effective radiosensitizing agent against breast cancer cells via a p21-dependent mechanism.

## Materials and Methods

### Compound

MLN4924 was a gift from Millennium Pharmaceuticals, Inc. (Cambridge, MA) [15]. The compound was dissolved in DMSO to make a 10 mM stock solution and kept in −20°C before use.

### Cell Culture

Breast cancer lines, including SK-BR-3, MCF-7, ZR-75-1 and T-47D were obtained from ATCC and were grown in DMEM (Gibco) with 10% fetal bovine serum.

### Radiation exposure and clonogenic assay

Cells were seeded in 60-mm dishes at proper cell densities in duplicate and exposed to different doses of radiation (Philips RT250, Kimtron Medical) after 24 hrs pre-treatment with MLN4924, followed by incubation at 37°C for 7 to 11 days. Survival curves were fitted using the linear-quadratic equation, and the mean inactivation dose was calculated [28]. Irradiations were performed in the Experimental Irradiation Core of the University of Michigan Cancer Center.

### siRNA knockdown of p21

The siRNA oligonucleotide target p21 expression is as follows: si-p21 (5′-GUGGACAGCGAGCAGCUGAUU-3′), and scrambled control siRNA (siCONT: 5′-AUUGUAUGCGAUCGCAGACUU-3′). The oligoes were purchased from Dharmacon (USA). Cells were transfected with siRNA using Lipfectamine 2000 and split 48 hrs later. One portion was used for clonogenic assay and the other portion for immunoblotting (IB) [29].

### Western blotting

Cells were exposed to various treatments and harvested for Western blotting as described [29] using antibodies against CDT1, WEE-1, CUL1 (Santa Cruz Biotechnology), ORC-1, p21, p27 (BD Biosciences), NOXA (Calbiochem), γ-H2AX (MILLPORE), PARP, Caspase-3, BIM-EL, phospho-CHK1 (S345), phospho-CHK2 (T68), total CHK1 and CHK2 (Cell Signaling), and β-actin (Sigma).

### FACS (Fluorescence-activated Cell Sorting) analysis

Cells were treated with MLN4924, or exposed to radiation alone or in combination. Cells were harvested 24 or 48 hrs post radiation and analyzed by flow cytometry [29].

### DNA fragmentation assay

Cells were seeded in 100-mm dishes at 0.5×10^6^ cells per dish, and pretreated next day with DMSO (control) or MLN4924 (100 nM) for 6 hrs before being exposed to radiation (6 Gy). The cells were harvested for 48 hrs post radiation by scraping, pelleted and lysed in 600 µl of lysis buffer (5 mM Tris-HCl, pH 8, 20 mM EDTA and 0.5% Triton X-100). The fragmented DNAs in the supernatant after 14,000 rpm centrifugation were extracted with PCI (Phenol/Chloroform/Isopropanol) (Fisher) and precipitated with ethanol, followed by electrophoresis in a 1.8% agarose gel [30].

### Statistical analysis

ANOVA were used with SPSS (Statistical Product and Service Solution) software for statistical comparisons involving multiple groups, followed by SNK post hoc test to determine significance of each two group (*p*<0.05). Paired or unpaired two-tailed Student *t* test was performed in comparison between two groups, using SAS software.

## Results

### MLN4924 sensitized breast cancer cells to radiation

**Figure 1:**
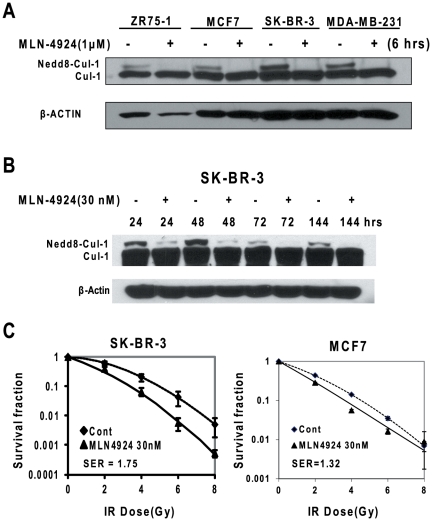
MLN4924 sensitizes breast cancer cells to radiation. (**A&B**) Cullin deneddylation: Subconfluent breast cancer cells were treated with MLN4924 at indicated concentrations or for indicated time periods, followed by immunoblotting (IB) with β-actin as a loading control. (**C**) Radiosensitization by MLN4924: SK-BR-3 or MCF7 cells were seeded in 60-mm dishes in duplicate and treated with MLN4924 (30 nM) and radiation at various doses. The colonies with more than 50 cells were counted after 7–11 days. Surviving fraction was calculated as the proportion of seeded cells following irradiation to form colonies relative to that of untreated cells (mean ± SEM) (n = 3 for SK-BR-3 cells and n = 2 for MCF7 cells). SER was calculated as the ratio of the mean inactivation dose under untreated control conditions divided by the mean inactivation dose after MLN4924 treatment.

Our most recent study showed that MLN4924 is a potent radiosensitizing agent against pancreatic cancer cells [27]. Here we extended the observation to breast cancer cells. We first determined the efficacy of MLN4924 in inactivation of SCF E3 ligases as reflected by cullin deneddylation in multiple breast cancer cells. As shown in Figure 1A, in all tested breast cancer lines, a portion of cullin-1 was neddylated, and cullin-1 neddylation was completely inhibited after 6 hrs of exposure to MLN4924 at 1 µM (Figure 1A). We next measured a time-dependent inhibition of cullin-1 neddylation with a much lower concentration of MLN4924 and found that MLN4924 at 30 nM caused a remarkable inhibition of cullin-1 neddylation at 24 hrs, and a complete inhibition at 72 hrs and thereafter up to 144 hrs (Figure 1B). We therefore used 30 nM of MLN4924 in our radiosensitization experiment and found that MLN4924 at such a low concentration caused a remarkable radiosensitization in SK-BR-3 cells with a sensitivity enhancement ratio (SER) of 1.75 (Figure 1C). Similar observation was made in MCF7 cells, but to a lesser extent with a SER of 1.32 (Figure 1C). Thus, we conclude that MLN4924 is a potent radiosensizer against breast cancer cells.

### MLN4924 radiosensitization is associated with enhanced G2/M arrest and apoptosis

**Figure 2:**
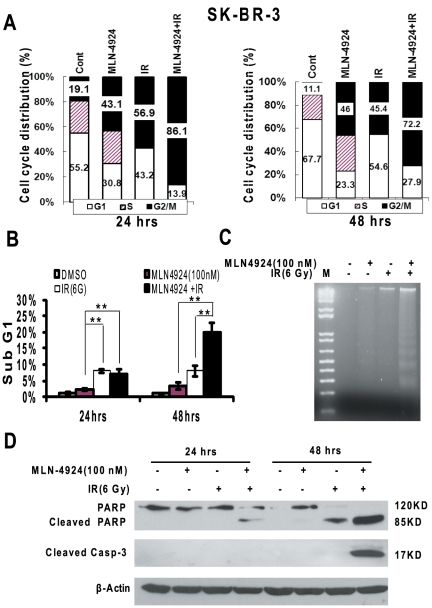
MLN4924 alters cell cycle profile and induces apoptosis. SK-BR-3 cells were treated with MLN4924 at 100 nM for 24 hrs or 48 hrs (**A&B**), alone or in combination with radiation (6 Gy), followed by FACS analysis for cell cycle profile (**A**), sub-G1 population for apoptosis (**B**), DNA fragmentation (48 hrs treatment) (**C**) and Western blotting with indicated antibodies (**D**). Shown is mean (**A**) or mean ± SEM (**B**), (n = 3): **, *p*<0.01. One representative result is shown.

**Figure 3:**
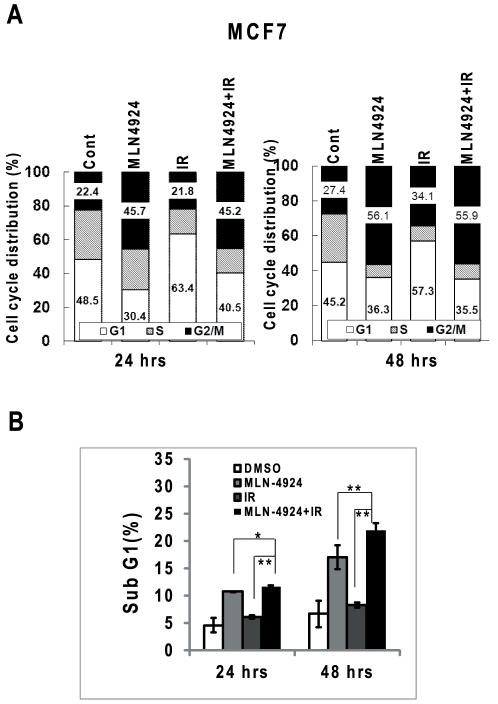
MLN4924 enhances radiation-induced apoptosis. MCF7 cells were treated with MLN4924 at 100 nM for 24 hrs or 48 hrs (**A&B**), alone or in combination with radiation (6 Gy), followed by FACS analysis for cell cycle profile (**A**) and sub-G1 population for apoptosis (**B**). Shown is mean (**A**) or mean ± SEM (**B**), (n = 3): **, *p*<0.01. One representative result is shown.

To determine the nature of MLN4924 radiosensitization, we performed cell cycle profile of two breast cancer cell lines treated with MLN4924, radiation, alone or in combination using FACS analysis. As shown in Figure 2A (left panel), treatment with MLN4924 or radiation for 24 hrs remarkably arrested SK-BR-3 cells (harboring a mutant p53) [31] at the G2/M phase of cell cycle (43% or 57% vs. control at 19%, respectively). The combinational treatment further enhanced G2/M arrest with 86% of population arrested in the G2/M. The enhanced G2/M arrest persisted up to 48 hrs (Figure 2A, right panel). Furthermore, FACS analysis also showed that radiation, but not MLN4924, induced apoptosis (as reflected by sub-G1 population) after 24-hrs treatment, which was not enhanced by MLN4924 at 24-hrs, but was enhanced at 48-hrs time point (Figure 2B). Consistenly, significant induction of apoptosis was seen in SK-BR-3 cells treated with radiation-MLN4924 combination, as demonstrated by enhanced DNA fragmentation (Figure 2C) as well as PARP cleavage and caspase-3 cleavage/activation (Figure 2D). However, in wild type p53-containing MCF7 cells [31], MLN4924 induced growth arrest at the G2/M phase of cell cycle at either 24-hrs (45.7% vs. 22.4%) or 48-hrs (56.1% vs. 27.4%) time point, whereas radiation induced G1 arrest in both time points (63.4% vs. 48.5% and 57.3% vs. 45.2%, respectively) (Figure 3A). Compared to MLN4924 treatment alone, MLN4924-radiation combination had little effect on cell cycle profile (Figure 3A), but did cause a significant induction of apoptosis (Figure 3B). These results suggested that radiation-induced disruption of cell cycle progression in SK-BR-3 cells and apoptotic cell death in both SK-BR-3 and MCF7 cells, can be further enhanced by MLN4924.

### Blockage of apoptosis failed to abrogate MLN4924 radiosensitization

**Figure 4:**
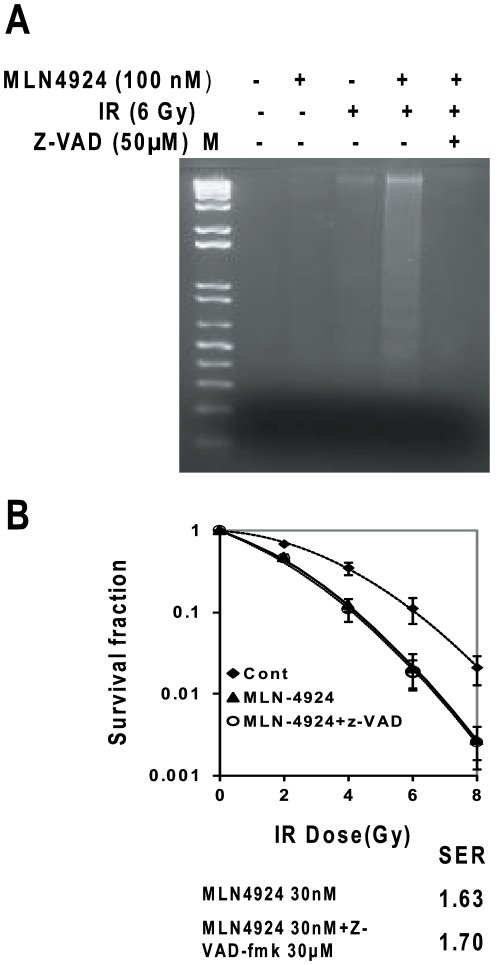
Blockage of apoptosis fails to abrogate MLN4924 radio sensitization. SK-BR-3 cells were treated with MLN4924 at 100 nM for 48 hrs, alone or in combination with radiation (6 Gy) in the absence or presence of pan caspases inhibitor, Z-VAD, followed by DNA fragmentation (**A**) or standard clonogenic assay (**B**). Shown is mean ± SEM (**B**) (n = 2).

Since our recent work showed that activation of caspases, followed by induction of apoptosis was causally related to radiosensitization by SMAC mimetic compound SM-164 in both breast and head and neck cancer cells [32], [33], we next determined potential causal role of caspase activation/apoptosis induction in MLN4924 radiosensitization in SK-BR-3. Surprisingly, although treatment of pan-caspase inhibitor, Z-VAD completely blocked apoptosis (Figure 4A), it had no effect at all on MLN4924-induced radiosensitization with SER remained at ∼1.7 (Figure 4B). These results clearly indicated that caspase activation and apoptosis induction was not causally related to MLN4924 radiosensitization.

### MLN4924 had little effect on radiation-induced DNA damage response, but caused minor delay in DNA repair

**Figure 5:**
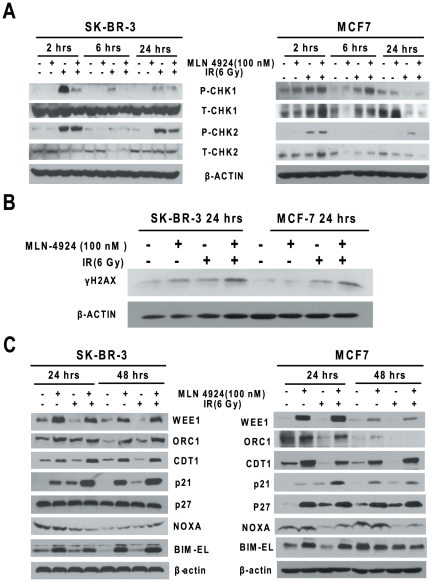
MLN4924-radiation triggers DNA damage response and induces accumulation of SCF E3 ligase substrates. Subconfluent SK-BR-3 and MCF7 cells were treated with MLN4924 (100 nM) or radiation (6 Gy) alone or in combination for indicated time periods up to 48 hrs, followed by IB analysis using indicated antibodies.

Since the major cellular effect of ionizing radiation is to cause DNA damage and trigger the DNA damage response [34], we, therefore, examined if MLN4924 treatment would enhance radiation-induced DNA damage and interfere with the DNA damage repair process. We first determined the DNA damage response upon MLN4924-radiation treatment by measuring phosphorylation of CHK1 and CHK2 and found that while radiation indeed caused phosphorylation/activation of CHK1 and CHK2 in both SK-BR-3 and MCF7 cells, MLN4924 had little, if any, enhancing effect (Figure 5A). In fact, MLN4924 treatment reduced radiation-induced CHK1 phosphorylation in SK-BR3 cells, consistent with a recent study in which MLN4924 suppressed CHK1 phosphorylation induced by DNA damage agents, such as UV and Cisplatin [35]. We next determined DNA double-strand breaks (DSBs) by measuring the overall levels of γ-H2AX protein at 24 hrs post exposure to radiation or MLN4924, alone or in combination. We found that while γ-H2AX levels increased after single treatment, the combination treatment caused a further increase in both breast cancer lines (Figure 5B). Thus, MLN4924 had little role in enhancing radiation-induced DNA damage response, but did delay DNA repair process, which could contribute to its radiosensitizing effect.

### MLN4924 selectively induced accumulation of p21, but not other SCF E3 ligase substrates, known to regulate DNA damage response and apoptosis

To identify potential mediator(s) of MLN4924 radiosensitization, we measured the levels of proteins known to be a) the substrates of SCF E3 ligases and b) involved in the regulation of cell cycle progression (e.g. p21, p27 and WEE1), DNA damage response (e.g. CDT1 and ORC1), and apoptosis (e.g. NOXA and BIM-EL) [2], [24], [36]. We found that as expected, the levels of cell cycle regulators, such as p21 and WEE1, DNA licencing proteins CDT1 and ORC1, and apoptosis inducer BIM-EL, but not NOXA, increased substantially upon treatment with MLN4924, but not radiation (Figure 5C). Unlike what were observed in pancreatic cancer cells [27], the MLN4924-radiation combination did not further increase the levels of CDT1 and WEE1, but did caused a further increase of p21 in both SK-BR-3 and MCF7 cells (Figure 5C). Thus, p21 is the only SCF E3 ligase substrate, among all tested substrates, that was induced by either MLN4924 or radiation, leading to a higher level of accumulation upon combination.

### G2/M arrest and radiosensitization by MLN4924 is partialy inhibited upon p21 siRNA knockdown

**Figure 6:**
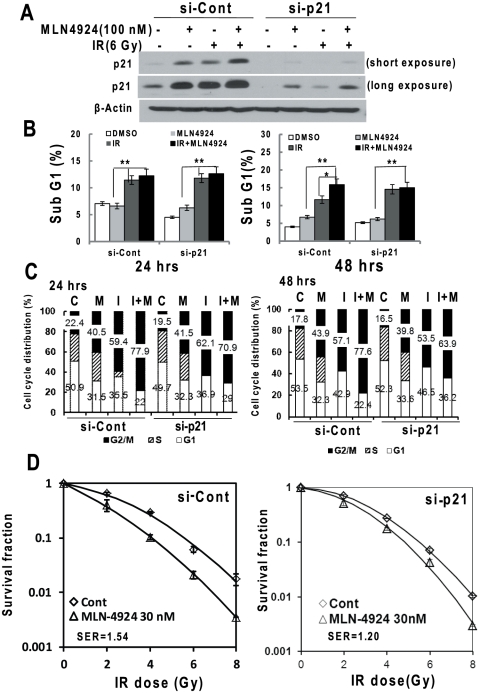
siRNA knockdown of p21 partially abrogates MLN4924 radiosensitization. SK-BR-3 cells were transfected with siRNA oligonucleotides targeting p21, along with scrambled control siRNA (siCont). Forty-eight hrs later, one portion of cells was subjected for Western blotting (**A**), the other portion was subjected to treatment by MLN4924, radiation or in combination for 24 or 48 hrs, followed by FACS analysis for sub-G1 apoptotic population (**B**, mean ± SD, n = 2) or cell cycle profile (**C**, mean number shown, n = 2, C: control, M: MLN4924, I: IR, M+I: MLN4924+IR). Still other portion was plated for clonogenic assay (**D**, mean ± SD, n = 3).

Given the fact that MLN4924 enhanced G2/M arrest and apoptosis by radiation and caused radiosensitization in SK-BR-3 cells (Figure 2), and that p21 was the only SCF E3 substrate whose accumulation by MLN4924 or radiation was further enhanced by the combination (Figure 5C), we determined whether accumulated p21 was causally related to G2/M arrest, apoptosis, and radiosensitization by a siRNA mediated knockdown experiment. Upon p21 knockdown (Figure 6A), degree of apoptosis induced by MLN4924-radiation combination was not changed (Figure 6B), as compared to the scrambled si-control. The G2/M arrest, however, was partially reversed from 77.9% to 70.9% at 24 hrs and 77.6% to 63.9% at 48 hrs (Figure 6C), indicating that p21 accumulation contributes at least in part to substantial G2/M arrest. More importantly, MLN4924-induced radiosensitization were remarkably inhibited upon p21 knockdown with SER value reduced from 1.54 to 1.2 (Figure 6D). Taken together, these results clearly suggest a causal effect of p21 accumulation (upon SCF E3 inactivation by MLN4924) on MLN4924-induced G2/M arrest and radiosensitization in SK-BR-3 cells. On the other hand, the incomplete rescue of MLN4924-triggered G2/M arrest and radiosensitization by p21 knockdown might be attributable to a) incomplete p21 elimination (Figure 6A), b) accumulation of other yet-to-be identified SCF E3 substrates, and/or c) delayed DNA repair process (Figure 5B), which was not rescued by p21 knockdown (data not shown).

## Discussion

Breast cancer is one of the most frequently diagnosed cancers and the leading cause of cancer death among women around world [37]. Although significant advance in the development of targeted therapy for breast cancer [38], radiotherapy remains to be an effective option in achieving the local control of the disease, particularly after conservative surgery [39], [40]. However, development of radioresistance in breast cancer cells limits the curable potential and contribute to local recurrences [41], [42]. Thus, development of radiosensitizing agents against breast cancer is in high demanding for the improvement of therapeutic outcome. To pursue this end, we have recently found that radioresistant breast cancer cells can be sensitized to radiation by a small molecule SMAC mimetic compound SM-164 [32]. The mechanism of SM-164 radiosensitization was mediated by activation of caspases and induction of apoptosis via inhibiting cellular inhibitor of apoptosis proteins, cIAP-1 and XIAP [32].

In this study, we tested our working hypothesis that inactivation of SCF E3 ubiquitin ligases by MLN4924, a small molecule inhibitor of NAE [15], would sensitize breast cancer cells to radiation, given the fact that some of SCF E3 components, such as β-TrCP and SAG, were overexpressed in breast cancer cells which was associated with chemo- or radio-resistance [24], [43]. Indeed, we showed here that MLN4924 is a potent radiosensitizing agent against breast cancer cells. Mechanistic study revealed that although MLN4924 enhanced radiation-induced apoptosis, apoptosis induction appeared not to be causally related, since complete blockage of apoptosis by a pan-caspase inhibitor Z-VAD had no effect on MLN4924 radiosensitization. Unlike the mechanism by which MLN4924 sensitized pancreatic cancer cells to radiation via causing accumulation of CDT1 and WEE1 to trigger DNA damage response/aneuploidy and G2/M arrest, respectively [27], MLN4924-induced radiosensitization in breast cancer cells appeared not to involve CDT1 or WEE1. Instead, it is mediated at least in part by p21, as demonstrated by 1) either MLN4924 or radiation induced p21 level, which was further induced by the combination, and 2) p21 knockdown partially reversed G2/M arrest and largely abrogated MLN4924 radiosensitization. Thus, p21 mediated not only MLN4924-induced senescence in HCT116 colon cancer cells and H1299 lung cancer cells [19], but also MLN4924-induced radiosensitization in SK-BR-3 and MCF7 breast cancer cells.

p21 is a universal inhibitor of cyclin dependent kinases and a well-known target of p53 [44], [45], [46]. Activation of p21 mediates p53 induced growth arrest at both G1 and G2 phases of cell cycle [47], [48]. In response to DNA damage and mitogen starvation, p21 directly interacts and inhibits cyclin A- and cyclin B-associated CDK1 to block G2/M progression [49], [50], [51], [52]. In this study, induction of p21 by radiation could be p53 dependent, as seen in MCF7 cells harboring a wild type p53 or p53-independent in SK-BR-3 cells with a mutant p53 [31], through other transcription factors such as SP1 [53]. Induction of p21 by radiation has been seen in many other cancer cells, mainly at the transcriptional levels [54], [55], whereas induction of p21 by MLN4924 was likely mediated by blockage of its degradation, since p21 is well-known substrate of SCF^Skp2^ E3 ubiquitin ligase [2], [56]. We observed that in SK-BR-3 cells, either radiation or MLN4924 caused a significant G2/M arrest, which was further enhanced by their combination in which 86% and 72% of cell population was arrested in the G2/M phase of cell cycle after the treatment for 24 hrs or 48 hrs, respectively (Figure 2A). Partial rescue of G2/M arrest by p21 knockdown (Figure 6C) strongly suggests a causal involvement, at least in part, of p21 in the process.

It appeared paradoxical that cells arrested in the G2/M phase became most sensitive to radiation, given the conventional wisdom that G2 arrest will allow cells more time to repair damaged DNA, thus protecting cells. The fact that consistent higher level of γH2AX, a well-known marker for DNA damage response/repair, in combination group than that in radiation alone group suggests that extended G2 arrest, likely resulting from prolonged process of DNA damage repair, could contribute to MLN4924 radiosensitization. In fact, radiosensitiation of H460 and A549 lung cancer cells by a tryptamine compound, and of PC-3 prostate cancer cells by genistein also involved p21 induction and G2/M arrest [57], [58].

In summary, our study revealed a potent radiosensitizing activity of MLN4924, a small molecule inhibitor of SCF E3 ligases, in breast cancer cells, in addition to our recent observation made in pancreas and lung cancer cells [27]. Different mechanism of action for MLN4924 radiosensitization (p21 in breast cancer cells vs. CDT1 and WEE1 in pancreatic cancer cells) reflected a cell-line dependent response to inactivation of SCF E3 ligases. Taken together, our studies provide an appealing piece of evidence for future development of MLN4924 as a novel radiosensitizing agent against various human cancers, including breast cancers, with activated SCF E3 ligases.
